# Are You Being Served? A Survey of the Opinions of G.Ps. Referring Patients to the Bristol Dental Hospital

**Published:** 1989-02

**Authors:** B G H Levers, C M Scully, R W Matthews

**Affiliations:** University Department of Oral Medicine, Surgery and Pathology, Bristol Dental Hospital and School, Lower Maudlin Street, Bristol BS1 2LY; University Department of Oral Medicine, Surgery and Pathology, Bristol Dental Hospital and School, Lower Maudlin Street, Bristol BS1 2LY; University Department of Oral Medicine, Surgery and Pathology, Bristol Dental Hospital and School, Lower Maudlin Street, Bristol BS1 2LY


### Are you being served?

### A survey of the opinions of general medical practitioners who refer patients to Bristol Dental Hospital

**B G H Levers BSc, MSc, PhD**
**C M Scully BSc, BDS, MBBS, PhD, FDSRCPS, MRCPath**
**R W Matthews BDS, PhD, MDS**
University Department of Oral Medicine, Surgery and Pathology, Bristol Dental Hospital and School, Lower Maudlin Street, Bristol BS1 2LY

We recently carried out a survey of general medical practitioners regarding their opinions of the service they receive for patients whom they refer to Bristol Dental School and Hospital (BDH).

### METHOD

Anonymous questionnaires were circulated in Spring 1987 by the Family Practitioner Committee to 523 general medical practitioners in the Avon Family Practitioner catchment area (Table 1). Questions were posed as to the frequency of referral, the departments or units to which patients were referred and the practitioner's general opinion as to the service provided. A section was also devoted to eliciting specific criticism of the service provided, if any.

### RESULTS

Replies were received from 334 medical practitioners (63.9% response) (Table 1).

**Table 1**

**Details of survey**

| | *General Medical Practitioners No.* | % |
|---|---|---|
| No. of questionnaires | 523 | |
| Replies | 334 | 63.9 |
| Practitioners referring patients to BDH | 210 | 62.9 of replies |

Medical practitioners who referred patients to BDH accounted for 210 (62.9%) of the respondents.

From Table 2 it can be seen that the vast majority of referrals from medical practitioners is on an 'occasional' basis only.

**Table 2**

**Frequency of referral**

| | *No.* | % |
|---|---|---|
| Weekly | — | — |
| Fortnightly | 1 | 0.5 |
| Monthly | 9 | 4.3 |
| Occasionally | 200 | 95.2 |

Table 3 shows that most departments receive referalls, the exception being the 'Preventive'. The Oral Medicine and Oral Surgery units receive the biggest share of referrals.

**Table 3**

**Units or departments to which patients are referred**

| | *No.* | % |
|---|---|---|
| 1. Oral Surgery | 136 | 64.8 |
| 2. Casualty/Diagnosis | 99 | 47.1 |
| 3. Oral Medicine | 96 | 45.7 |
| 4. Periodontology | 9 | 4.3 |
| 5. Prosthetics | 7 | 3.3 |
| 6. Conservation | 6 | 2.9 |
| 7. Orthodontics | 6 | 2.9 |
| 8. Children's | 4 | 1.9 |
| 9. Preventive | — | — |

Nearly all practitioners referred patients to more than one department which accounts for the overall total of 364 departmental or unit referrals by GMPs.

Table 4 shows the overall opinion of practitioners as to the service provided. Some three quarters of referring GMPs (75%) ranked the service as "good" or "excellent": nearly 18% ranked it "excellent". Some 15% felt the service was "average". Only 5 out of 193 (2.6%) GMPs who expressed an opinion, were dissatisfied, in general terms, with the service provided.

**Table 4**

**Overall opinion of BDH service**

| | *No.* | % |
|---|---|---|
| Excellent | 37 | 17.6 |
| Good | 121 | 57.6 |
| Average | 31 | 14.8 |
| Poor | 3 | 1.4 |
| Bad | 1 | 0.5 |
| Not stated | 17 | 8.1 |

Table 5 shows the numbers of complaints and the main categories in which complaints were made.

Further analysis showed that 22 of the medical practitioners who were otherwise reasonably satisfied, felt strongly enough to register 25 complaints about particular fields of service. Together with the 5 who considered that the service was 'poor' or 'bad' overall (4 complaints), this amounts to a total of 26 GMPs (12.4%) who registered 29 causes for complaint. No opinion as to the overall standard of service was given by 17 GMPs (8.1%).

**Table 5**

**Main areas of criticism of BDH service**

| | *No.* | % |
|---|---|---|
| Long appointments' waiting list | 6 | 2.9 |
| Long operations' waiting list | — | — |
| Presence of students | 1 | 0.5 |
| Unhelpful staff | — | — |
| Poor telephone communications | 2 | 1.0 |
| Patient not seen by consultant to whom referred | 1 | 0.5 |
| Unsatisfactory correspondence | 4 | 1.9 |
| Miscellaneous other* | 16 | 7.6 |

* Includes comments about

| | |
|---|---|
| No emergency service provided | (12) |
| Insufficient knowledge of departments | (2) |
| Too frequent recall | (1) |
| Inadequate supervision of students | (1) |

The usual reasons given by the 124 practitioners who did not refer patients to BDH were that:-

a. they were unaware that the service existed
b. there were no weekend referrals
c. they referred patients elsewhere

Only 2 did not refer patients to BDH because of some past dissatisfaction. Both of these were stated to be because of a lack of after-hours service.

### CONCLUSION

Whilst most practitioners who expressed an opinion were more than satisfied overall with the service which they receive from BDH, the incidence of certain criticisms shows that there are a number of important areas where improvements in the service can be made.

A new telephone exchange is currently being installed for 1988 and this, together with new Consultant clinics and a new Primary Care Unit, as well as improved organisation should help communications and reduce waiting lists for appointments—two of the major problem areas. Practitioners should, however, appreciate that there has been chronic shortage of medical secretaries and records staff and that there are increasing cutbacks in funding of many acute services in the country. In 1982 BDH was run on 1.5 medical secretaries, though this is now 4. The total number of outpatient attendances is about 105,000 per year.

Furthermore, we must provide the service in those areas not the remit of general dental practitioners. We provide a 24 hour Accident and Emergency service for serious problems such as trauma, haemorrhage and infection. Provision of a 24 hour toothache service is explicitly not the function of the hospital dental service and it would only be justified on the grounds of the contribution to undergraduate training. Indeed, the rise in personal violence is already throwing an increasing burden on these services (Shepherd et al., 1986).

### REFERENCES

SHEPHERD, J. P., SHAPLAND, M., IRISH, M., SCULLY, C., LESLIE, I., PARSLOE, P. *et al.* Higher rates of assault in the unemployed. *Lancet ii*: 1038, 1986.

